# Protection of Tight Junctional Complexes between hCMEC/D3 Cells by Deep-Sea Fibrinolytic Compound FGFC1

**DOI:** 10.3390/md22080341

**Published:** 2024-07-26

**Authors:** Xiaozhen Diao, Hui Han, Haoyu Sun, Haixing Zhang, Wenhui Wu

**Affiliations:** 1 Department of Marine Bio-Pharmacology, College of Food Science and Technology, Shanghai Ocean University, Shanghai 201306, China; xzdiao@shou.edu.cn (X.D.); m220300899@st.shou.edu.cn (H.H.); m230351101@st.shou.edu.cn (H.S.); d230300097@st.shou.edu.cn (H.Z.); 2 Putuo Sub-Center of International Joint Research Center for Marine Biological Sciences, Zhoushan 316100, China; 3 Marine Biomedical Science and Technology Innovation Platform of Lin-gang Special Area, Shanghai 201306, China

**Keywords:** FGFC1, deep-sea fibrinolytic compounds, tight junctional complexes, blood–brain barrier, thrombolytic therapy

## Abstract

Tight junctional complexes (TJCs) between cerebral microvascular endothelial cells (CMECs) are essential parts of the blood–brain barrier (BBB), whose regulation closely correlates to the BBB’s integrity and function. hCMEC/D3 is the typical cell line used to imitate and investigate the barrier function of the BBB via the construction of an in vitro model. This study aims to investigate the protective effect of the deep-sea-derived fibrinolytic compound FGFC1 against H_2_O_2_-induced dysfunction of TJCs and to elucidate the underlying mechanism. The barrier function was shown to decline following exposure to 1 mM H_2_O_2_ in an in vitro model of hCMEC/D3 cells, with a decreasing temperature-corrected transendothelial electrical resistance (tcTEER) value. The decrease in the tcTEER value was significantly inhibited by 80 or 100 µM FGFC1, which suggested it efficiently protected the barrier integrity, allowing it to maintain its function against the H_2_O_2_-induced dysfunction. According to immunofluorescence microscopy (IFM) and quantitative real-time polymerase chain reaction (qRT-PCR), compared to the H_2_O_2_-treated group, 80~100 µM FGFC1 enhanced the expression of claudin-5 (CLDN-5) and VE-cadherin (VE-cad). And this enhancement was indicated to be mainly achieved by both up-regulation of CLDN-5 and inhibition of the down-regulation by H_2_O_2_ of VE-cad at the transcriptional level. Supported by FGFC1’s molecular docking to these proteins with reasonable binding energy, FGFC1 was proved to exert a positive effect on TJCs’ barrier function in hCMEC/D3 cells via targeting CLDN-5 and VE-cad. This is the first report on the protection against H_2_O_2_-induced barrier dysfunction by FGFC1 in addition to its thrombolytic effect. With CLDN-5 and VE-cad as the potential target proteins of FGFC1, this study provides evidence at the cellular and molecular levels for FGFC1’s reducing the risk of bleeding transformation following its application in thrombolytic therapy for cerebral thrombosis.

## 1. Introduction 

The BBB is a dynamic barrier separating neurons and brain tissue from the blood cycling system, not only preventing exogenous toxins and pathogens entering inside but also removing endogenous waste products [1]. The BBB is essential for maintaining the homeostasis in the microenvironment of the cerebral nervous system (CNS) [2], whose disruption or dysfunction, mainly caused by oxidative stress (OS), may lead to certain neurological diseases such as Alzheimer’s disease (AD), Parkinson’s disease (PD), stroke [3], etc. The function of the BBB is mainly achieved by its specifically selective and tightly controlled transcellular or paracellular permeability [4]. TJCs between CMECs include tight junctions (TJs), adhesive junctions (AJs), and zonula occludins (ZOs), which are responsible for the paracellular transport of micro-molecules or non-polar substances from the blood to the brain [5,6]. TJs contribute to maintaining the integrity and physiological function of the BBB by its structural and functional proteins, such as claudins (CLDNs) and occludin (OCLN). Among the CLDNs identified in the BBB (including CLDN-1, -3, -5, -12), CLDN-5 has been proven to be dominant in the tightness of TJs [7]. It was demonstrated that OCLN is abundant in the brain, and its down-regulation is associated with pathological breakdown of the BBB [8,9,10,11]. These TJ proteins are linked to the cytoskeleton by ZO-1 in the cell matrix [4], and AJs construct the attachment in paracellular gaps by VE-cad [12], both of which provide support for the structural integrity of TJs.

Marine natural products are known as a valuable source for drug discovery due to their unique chemical diversity and excellent biological activity [13,14]. Isolated from the metabolites of deep-sea fungus *Starchbotrys longispora* strain FG216 (or fungus *Stachybotrys microspora* strain IFO30018), FGFC1 (also known as SMTP-7, 2,5-Bis-[8-(4,8-dimethyl-nona-3,7-dienyl)-5,7-dihydroxy-8-methyl-3-keto-1,2,7,8-teraahydro-6*H*-pyran[a]isoindol-2-yl]-pentanoic acid, PubChem CID: 102219046) is an isoindolone alkaloid [15] which has a variety of biological activities including thrombolytic, anticancer, anti-inflammatory, and neuroprotective activities [16,17]. In 2010, Hashimoto et al. demonstrated FGFC1 as the first compound with both thrombolytic and anti-inflammatory activities in a mouse model of embolic infarction [18]. Apart from its preventing significant bleeding risk in a rat acute pulmonary thromboembolism model [19], FGFC1 has also been proven to have a wide therapeutic time window, remaining effective even after 3 h of ischemia. According to Shibata et al., FGFC1’s antioxidant properties are due to reducing the production of reactive oxygen species (ROS) to relieve brain damage after stroke [18]. The neuroprotective effect of FGFC1 was first demonstrated by Akamatsu et al., who showed that FGFC1 could reduce ischemic neuronal damage by inhibiting the OS response and matrix metalloproteinase-9 (MMP-9) activation in a mouse model of ischemic stroke [15].

FGFC1 has been proven to have significant therapeutic activity not only in cardiovascular and cerebrovascular diseases, but also neurological diseases, for example, cerebral thrombosis, atherosclerosis, and stroke [20,21]. However, studies on neurodegenerative diseases closely related to OS-caused injury to the BBB integrity and function, such as AD and PD, are still lacking. Based on the evidence from previous research, FGFC1 was indicated to alleviate the breakdown of the BBB by its antioxidant and anti-inflammatory activities. Therefore, focusing on TJCs in the BBB, this study aims to investigate the antioxidant protection by FGFC1 against H_2_O_2_-induced barrier dysfunction or disruption, and to elucidate the underlying mechanism. The results from this study confirmed the novel therapeutic activity of FGFC1 correlated with the BBB regulation related to TJCs as its new target proteins, which not only prolong its application in the treatment of neurodegenerative diseases, but also provide evidence at the molecular level for FGFC1 reducing the risk of bleeding transformation in its application in thrombolytic therapy for cerebral thrombosis.

## 2. Results 

### 2.1. The Construction of Barrier In Vitro Model in hCMEC/D3 Cells

**Figure 1:**
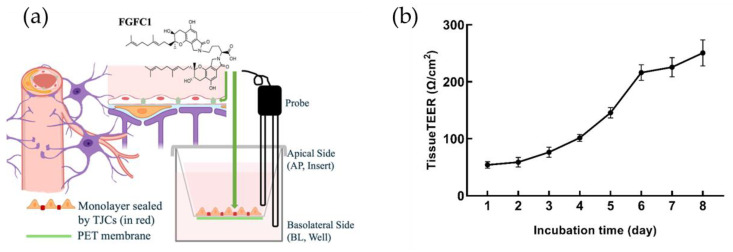
The construction of the hCMEC/D3-based in vitro barrier model confirmed by TEER assay. (**a**) hCMEC/D3 cells were reseeded on the PET membrane of the hanging inserts in 24-well microplates and the barrier function was measured by the probe of a Millicell ERS-2 system; (**b**) hCMEC/D3 cells successfully formed a monolayer from 6 to 8 days after the reseeding, when the tcTEER values of the model became stable around 210–265 Ω/cm^2^ (310.15 K). Relative tcTEER values are means ± SD, *n* = 6.

In this study, hCMEC/D3 cells successfully formed a barrier from 6 to 8 days after reseeding, when the tcTEER values of the in vitro model became stable around 210–265 Ω/cm^2^ (310.15 K) (Figure 1). The in vitro model is shown to present strong barrier function against H_2_O_2_-induced OS, which could be further confirmed by both the cells’ morphological characteristics and the tolerance to long incubation with H_2_O_2_, up to 4 h.

### 2.2. The H_2_O_2_-Induced Barrier Dysfunction of hCMEC/D3 Cells

**Figure 2:**
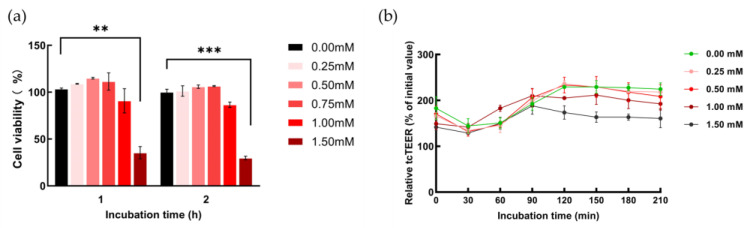
The H_2_O_2_-induced barrier dysfunction by TEER assay in hCMEC/D3 cells. (**a**) hCMEC/D3 cells treated with solutions with different concentrations of H_2_O_2_ were investigated by CCK-8; (**b**) the in vitro model of hCMEC/D3 was exposed to 3.5 h incubation with different concentration H_2_O_2_ solutions. Each cell viability or tcTEER value is indicated as mean ± SD, *n* = 6. Asterisks indicate a significant difference among solutions with various concentrations of H_2_O_2_ (** *p* < 0.025, *** *p* < 0.01), as determined by the Kruskal–Wallis test.

Various concentrations of H_2_O_2_ solutions were applied to the monolayer constructed by hCMEC/D3 cells for 4 h incubation to choose the appropriate concentration as the dysfunction inducer with no significant morphological damage, as observed by microscopy. Then, the influence of H_2_O_2_ on the viability of hCMEC/D3 cells was investigated using a CCK-8 kit after the addition of H_2_O_2_ (0.25, 0.50, 0.75, 1.00, 1.50 mM) to decide the optimal concentration without obvious cellular damage. It was suggested that the groups treated with the solutions with concentrations of H_2_O_2_ up to 1.50 mM showed potential cytotoxicity, with decreasing cell viability below 50% through 1–2 h of incubation (Figure 2a). Then, solutions with various concentrations of H_2_O_2_ (0.25, 0.50, 0.75, 1.00, 1.50 mM) were applied to the in vitro model constructed from hCMEC/D3 cells to choose the most appropriate one as the OS inducer to investigate the barrier dysfunction without irreversible damage, according to the related research on H_2_O_2_-induced barrier dysfunction by TEER assay [22] and our previous study on the decreasing tcTEER induced by H_2_O_2_ in an intestinal epithelial barrier in vitro model constructed by Caco-2 cells [23]. Combining the results by CCK-8, solutions with low concentrations of H_2_O_2_ (0.25, 0.50 mM) failed to decrease tcTEER values in hCMEC/D3 cells, while 1 mM H_2_O_2_ efficiently decreased the barrier function after 3.5 h of incubation (Figure 2b).

### 2.3. The Protection of TJCs in hCMEC/D3 Cells from Barrier Dysfunction by FGFC1

**Figure 3:**
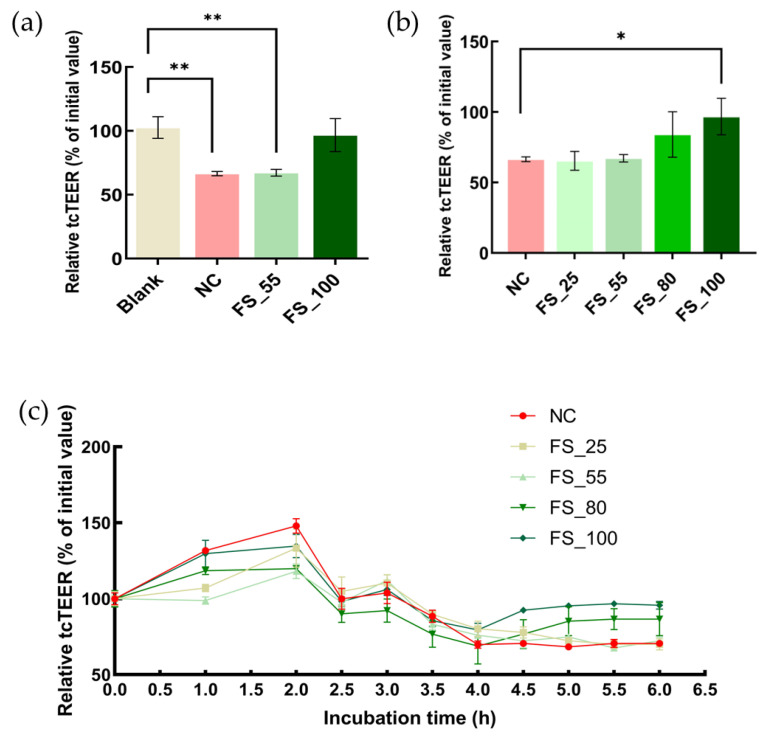
The protection of TJCs in hCMEC/D3 cells from barrier dysfunction (red) by FGFC1 at various concentrations (green series). (**a**) The maintenance of relative tcTEER values by FGFC1 solutions (FS_55, 100) against 1 mM H_2_O_2_ treatment (NC) or not (blank). (**b**,**c**) The relationship between relative tcTEER values and the concentration of FGFC1 solution (FS_25, 55, 80, 100) against 1 mM H_2_O_2_ treatment (NC). Monolayers constructed by hCMEC/D3 cells were pretreated with or without (NC) different concentrations (25, 55, 80, and 100 µM) of the FGFC1 solution for 2 h, followed by 1 mM H_2_O_2_ treatment for 4 h or not (blank). Relative tcTEER values are means ± SD, *n* = 6. Asterisks indicate a significant difference compared to blank (** *p* < 0.005), determined by the Mann–Whitney U test, or among various concentrations of the FGFC1 solutions (* *p* < 0.05), determined by the Kruskal–Wallis test.

The decreasing tcTEER values caused by 1 mM H_2_O_2_ in hCMEC/D3 cells were inhibited by the FGFC1 solution (2 h pretreatment before the H_2_O_2_ addition), with a dose-dependent effect, after 4 h incubation with H_2_O_2_ (Figure 3a,b). The tcTEER values finally decreased by 33.52% of the initial value, caused by 1 mM H_2_O_2_ treatment for 2 h. High concentrations of FGFC1 (80 or 100 µM) significantly maintained the tcTEER values (84.05% and 96.72%, respectively); this was not observed in the low-concentration groups (25 and 55 µM). The tcTEER values temporarily increased by the FGFC1 solutions or fresh medium started to decrease right after the H_2_O_2_ addition; the decreasing tcTEER values caused by H_2_O_2_ gradually recovered in all the groups and eventually reached a stable phase after 3 h incubation with H_2_O_2_ (Figure 3c).

### 2.4. The Alleviation by FGFC1 of TJC Depression Related to Barrier Dysfunction in hCMEC/D3 Cells

**Figure 4:**
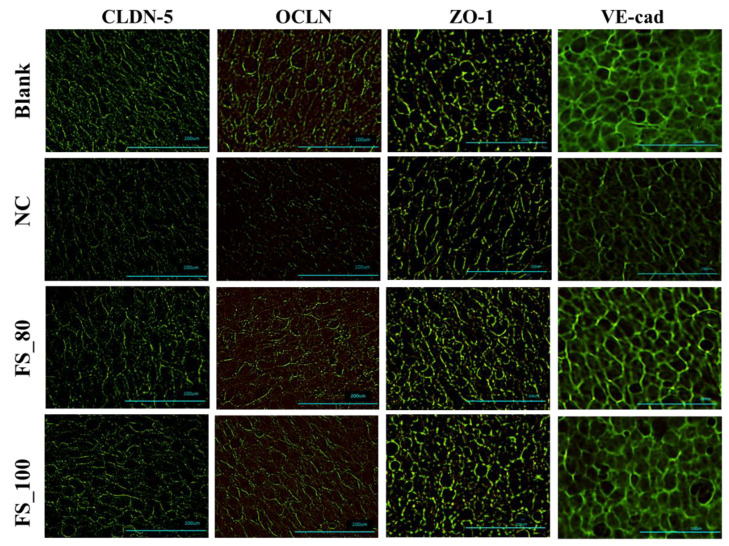
The protection by FGFC1 of the expression of TJC proteins including CLDN-5, OCLN, ZO-1, and VE-cad. Monolayers constructed by hCMEC/D3 cells were pretreated with different concentrations of the FGFC1 solution (80 and 100 µM) or without (NC) for 2 h, followed by 1 mM H_2_O_2_ treatment or not (blank) for 4 h. Images were observed by fluorescence microscopy. Each image is representative of 3 similar experiments. The scale bar is 200 µm.

**Figure 5:**
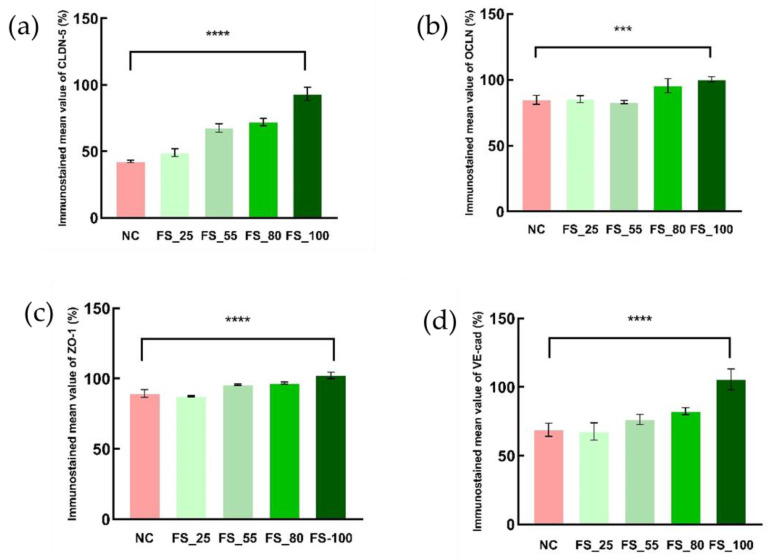
The enhancements in TJC protein expression in barrier dysfunction (red) caused by various concentrations of FGFC1 (green series). Four immunostained photomicrographs of CLDN-5 (**a**), OCLN (**b**), ZO-1 (**c**), and VE-cad (**d**) were separately randomly selected in each group and semi-quantitatively analyzed by ImageJ. Immunostained mean value is calculated by the mean integral optical density of each group relative to blank. Bars are means ± SD, *n* = 4. Asterisks indicate a significant difference between various concentrations of FGFC1 solutions (*** *p* < 0.01, **** *p* < 0.005), as determined by the Kruskal–Wallis test.

The H_2_O_2_-induced depression of the TJC proteins, including CLDN-5, OCLN, ZO-1, and VE-cad, was inhibited by pretreatment with 55–100 µM FGFC1, with a dose-dependent effect, during 4 h incubation with 1 mM H_2_O_2_ (Table 1). FGFC1 with a high concentration (80 or 100 µM) successfully maintained the expression of TJC proteins, with reference to the blank group (Figure 4). The inhibitory effects of FGFC1 on the depression of CLDN-5 and VE-cadherin were higher than those on OCLN and ZO-1 (Figure 5).

### 2.5. The Inhibitory Effects of FGFC1 on the Down-Regulation of TJCs’ mRNA Expression in hCMEC/D3 Cells 

**Figure 6:**
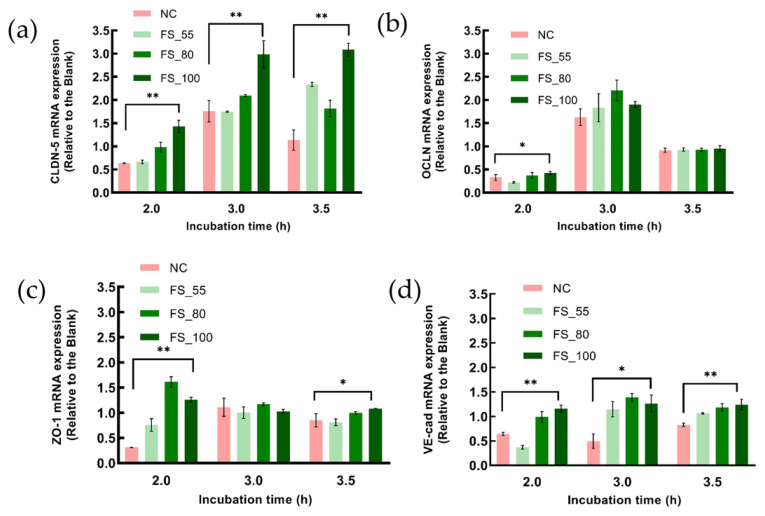
The influence of various concentrations of FGFC1 solution on the expression of TJC proteins (CLDN-5 (**a**), OCLN (**b**), ZO-1 (**c**), and VE-cad (**d**)) at the transcriptional level. Monolayers constructed by hCMEC/D3 cells were pretreated with various concentrations of FGFC1 solution (55, 80, and 100 µM) or without (NC) for 2 h, followed by 1 mM H2O2 treatment or not for 4 h. The total RNA was extracted from each group pretreated with the FGFC1 solution (2 h) at 2.0, 3.0, 3.5 h incubation time with H2O2 to investigate the mRNA level of each TJC protein. The mRNA expression relative to the blank is shown as mean ± SD, *n* = 6. Asterisks indicate a significant difference among various concentrations of FGFC1 solution (* *p* < 0.05, ** *p* < 0.025), as determined by the Kruskal–Wallis test.

The mRNA expression of CLDN-5 and VE-cad (Figure 6a,d) was continuously protected by the FGFC1 solution (2 h pretreatment before H_2_O_2_ addition) against the H_2_O_2_-induced down-regulation throughout 3.5 h treatment with 1 mM H_2_O_2_, with dose-dependent effects, which were not shown in the case of OCLN or ZO-1 (Figure 6b,c). The solutions with a high concentration of FGFC1 (80 or 100 µM) sufficiently up-regulated the mRNA expression of CLDN-5 at 2.0, 3.0, and 3.5 h of incubation, showing a relatively higher effect on the mRNA expression of VE-cad. Different from CLDN-5, FGFC1 solution only showed inhibitory effects on the H_2_O_2_-induced down-regulation of VE-cad.

### 2.6. Molecular Docking of FGFC1 to TJC Proteins

**Figure 7:**
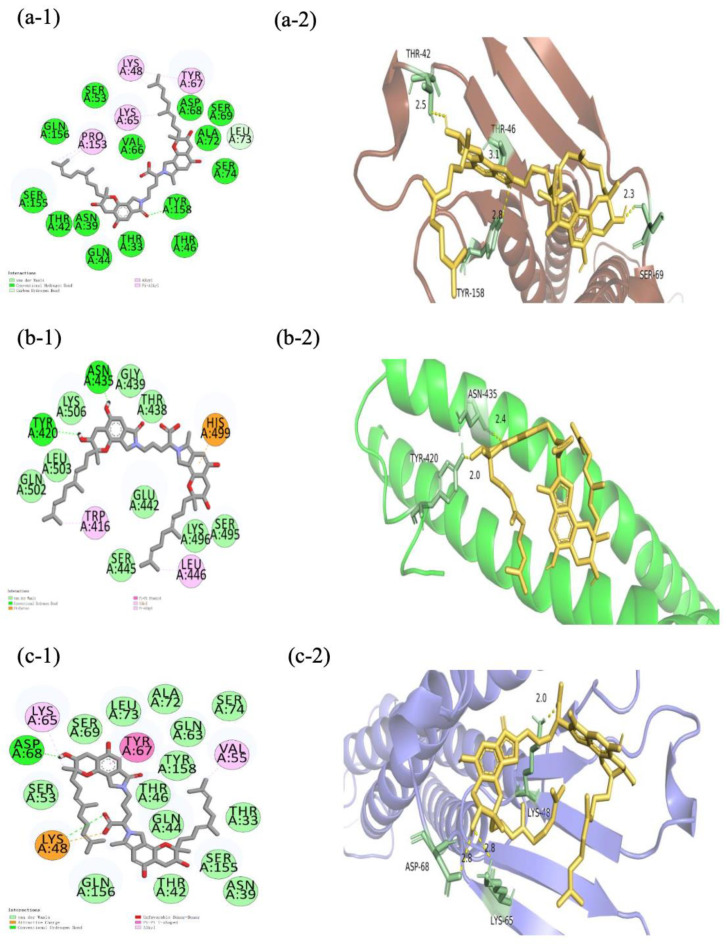
Schematic representation of the interaction between FGFC1 and the optimal conformation of CLDN-5, OCLN, and VE-cad. (**a-1**–**c-1**) The 3D model of FGFC1 docking CLDN-5, OCLN, and VE-cad (yellow: FGFC1, brown: CLDN-5, green: OCLN, purple: VE-cad). (**a-2**–**c-2**) Planar model of the binding sites of CLDN-5, OCLN, and VE-cad by FGFC1.

**Figure 8:**
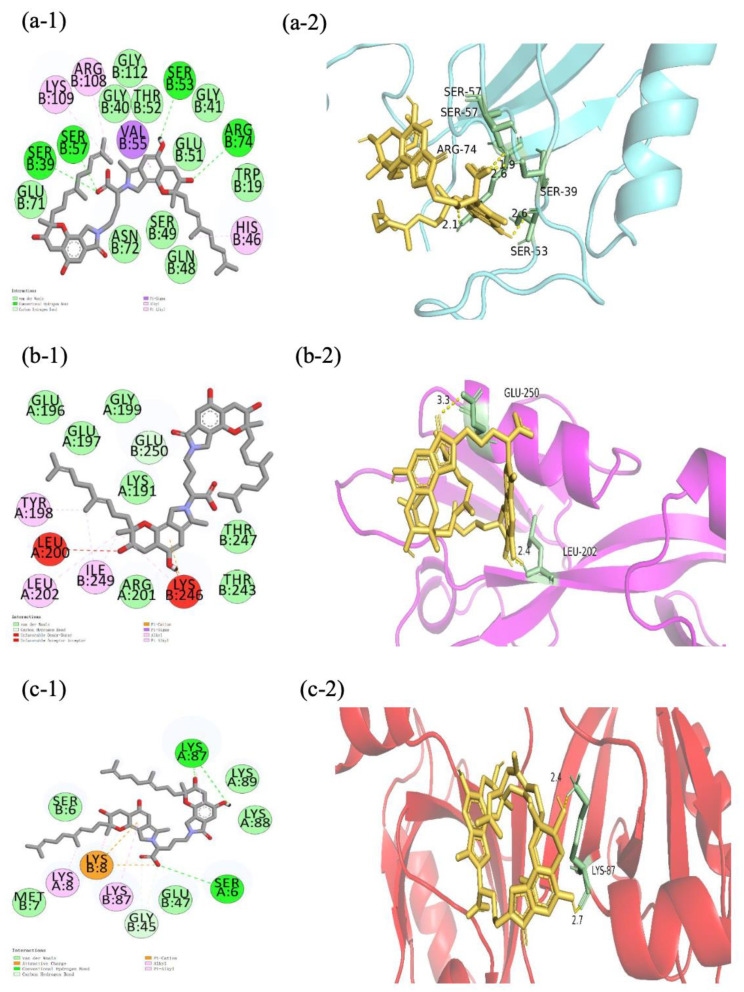
Schematic representation of the interaction between FGFC1 and the optimal conformation of ZO-1. (**a-1**–**c-1**) The 3D model of FGFC1 docking three domains of ZO-1 (yellow: FGFC1, blue: PDZ1, pink: PDZ2, red: PDZ3). (**a-2**–**c-2**) Planar model of the binding sites of PDZ1/2/3 of ZO-1 by FGFC1.

Considering the binding energy, the FGFC1-CLDN-5/OCLN/ZO-1/VE-cad conformations (−6.9, −6.1, (−7.7, −5.4, −5.0), and −0.72 kcal/mol, respectively) were selected among the top ten molecular docking conformations (Table 2), in order to further investigate the specificity and selectivity of ligand conformations. The three domains consisting of ZO-1 (PDZ1/2/3) underwent molecular docking with FGFC1 to investigate their binding energies separately (Figure 7). Among these TJC proteins, the FGFC1-CLDN-5 complex, FGFC1-VE-cad complex, and FGFC1-ZO-1(PDZ1) complex showed the highest binding energies, which indicates FGFC1’s preferential binding with them rather than the others (Figure 8).

## 3. Discussion 

OS-caused BBB disruption or dysfunction induced by ROS, such as H_2_O_2_, is associated with various neurological diseases or disorders including AD, Parkinson’s disease (PD), and stroke. The TEER value reflects the integrity and barrier function of TJCs in the BBB via the barrier in vitro model constructed by hCMEC/D3 cells; it could also be affected by cell status and viability. For example, fresh medium increased TEER values in hCMEC/D3 cells during the first 2 h of incubation right after the supplementation with FGFC1, which could probably be explained by its effect on the cells’ viability. Referring to the CCK-8 assay results, the decreasing cell viability of hCMEC/D3 cells caused by H_2_O_2_ is also responsible for the barrier dysfunction, which could be alleviated by pretreatment with the FGFC1 solution via its up-regulation of TJC proteins.

The protection of the barrier function of hCMEC/D3 by FGFC1 targeting TJC proteins, especially CLDN-5, OCLN, ZO-1, and VE-cad, was supported by the molecular docking for each target protein with reasonable binding energy. Three PDZ domains (PDZ1/2/3) of ZO-1 are known, with all playing essential roles in the ZO-1 structure responsible for TJs’ assembly and homeostasis [24]. PDZ1 and PDZ3 directly bind to CLDN-5 and JAM separately to give structure and support, while PDZ2 serves both binding functions. According to the binding energy of FGFC1 with each ZO-1 domain, the highest binding energy shown with PDZ1 would also support the preferable targeting of FGFC1 on CLDN-5. A relatively low binding energy of FGFC1-PDZ2/3 is suggested by FGFC1’s weak influence on ZO-1 itself. Compared to OCLN and ZO-1, CLDN-5 and VE-cad are proved to be more vulnerable to H_2_O_2_, whose OS-causing depression could be erased by FGFC1 at high concentration (100 μM). Given the effective up-regulation of CLDN-5 and the inhibition of H_2_O_2_-induced down-regulation of VE-cad by FGFC1 solutions, the maintenance of these two proteins was mainly achieved at the transcriptional level. The different levels of FGFC1’s regulation on these two proteins were mainly determined by their morphological distribution in TJCs. With its small molecular weight, FGFC1 prefers the paracellular route in TJCs, during which CLDN-5 at the top of the TJ structure would be preferentially targeted. Furthermore, combining the maintained barrier integrity and function of TJCs in hCMEC/D3 cells with FGFC1 shown in the TEER assay, CLDN-5 and VE-cad were indicated as the key regulatory factors for FGFC1 to regulate the barrier protection from H_2_O_2_-induced dysfunction, which is consistent with the essential roles of specific TJC proteins in BBB regulation previously reported. 

**Figure 9:**
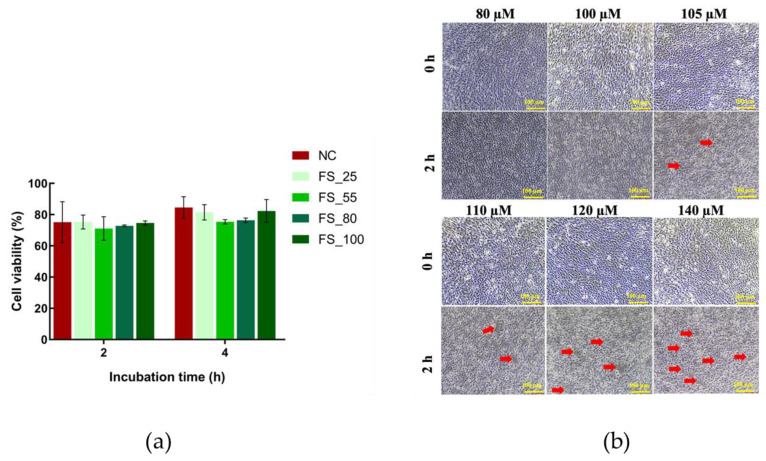
The optimization of the concentrations of FGFC1 solutions. (**a**) hCMEC/D3 cells pretreated with different concentrations (25, 55, 80, 100 µM) of FGFC1 (FS_25/55/80/100) followed by 2 and 4 h incubation with H_2_O_2_ were investigated by CCK-8. Each cell viability value is indicated as mean ± SD, *n* = 6. (**b**) The barriers constructed by hCMEC/D3 cells treated with various concentrations of FGFC1 solution, whose morphology characteristics were observed under the microscopy. Red arrows refer to the enlarged interval induced by the high concentrations of FGFC1 solutions. Each image is representative of 3 similar experiments. The scale bar is 100 µm.

Although the deep-sea-derived fibrinolytic compound FGFC1 has already been previously well investigated for its thrombolytic effects as a novel fibrinolytic compound, its effect on the concurrent ischemia/reperfusion-inducing BBB dysfunction or disruption remains under studied. Since FGFC1 is indicated to be a promising thrombolytic candidate, exhibiting significant fibrinolytic and anti-coagulation effects, the EC_50_ of its thrombolytic effect should also be taken into consideration in the study of its protection of the BBB. According to our previous study [21], the EC_50_ of FGFC1 fibrinolytic activity is 115.0 μM, around which the concentrations of FGFC1 were selected in this study. Furthermore, the optimization of the FGFC1 concentration was conducted by both the barrier morphology characteristics and CCK-8 assay of hCMEC/D3 cells. FGFC1 solutions with concentrations higher than 100 µM showed obvious damage to the barrier constructed by hCMEC/D3 cells following 2.0 h of treatment (see Figure 9b). According to the CCK-8 assay, the 100 µM FGFC1 solution already showed a decrease in cell viability (without potential cytotoxicity) during 4 h incubation with H_2_O_2_ (see Figure 9a). 

Since FGFC1 has already been reported to prevent significant bleeding risk in a rat acute pulmonary thromboembolism model [16], this article mainly provides evidence regarding the key factors of the BBB structure and function (TJCs), especially CLDN-5 and VE-cad. Still, in vivo experiments involving ischemia/reperfusion animal models should be further conducted to give more convincing evidence of the protection by FGFC1 on the BBB against the dysfunction or disruption caused by ischemia/reperfusion injury during thrombolytic therapy. This is the first report on the protection against H_2_O_2_-induced barrier dysfunction or disruption by the novel deep-sea fibrinolytic compound FGFC1, as well as its thrombolytic effect. With CLDN-5 and VE-cad as the potential target proteins of FGFC1, this study provides the regulatory factors for FGFC1 reducing the risk of bleeding transformation following its application in thrombolytic therapy for cerebral thrombosis.

## 4. Materials and Methods 

### 4.1. Chemicals and Reagents

Endothelial cell medium (ECM; Sciencell, Carlsbad, CA, USA) was used for the cell cultures. Rabbit polyclonal anti-OCLN, ZO-1 and Fluorescein isothiocyanate isomer Goat anti-Rabbit IgG were purchased from Proteintech (Wuhan, China). Rabbit polyclonal anti-CLDN-5 was purchased from Invitrogen (ThermoFisher, Waltham, MA, USA). Rabbit polyclonal anti-VE-cadherin was purchased from Abbkine (Wuhan, China). Horseradish peroxidase-conjugated Goat anti-Rabbit IgG was purchased from Affinity (Bioscience, Harvard, CA, USA). The various concentrations of diluted H_2_O_2_ solutions (Nanjing reagent, Nanjing, China) used were diluted by Dulbecco’s modified Eagle’s medium (DMEM, Procell, Wuhan, China) in this study.

### 4.2. The Preparation of the FGFC1 Solutions

Lyophilized FGFC1 powder and NaHCO_3_ (1:0.3) were dissolved in 0.9% NaCl for 10 min at room temperature, and then, stored at −80 °C to be used. The FGFC1 solutions were made by dilution with ECM as different concentrations, including 25, 55, 80, and 100 μM (FS_25, 55, 80, 100), right before the addition experiments.

### 4.3. Cell Cultures

Human cerebral microvascular endothelial cell-line (hCMEC/D3, Meisen, Zhejiang, China) cells were cultured with ECM at 37 °C in a 5% CO_2_ incubator. hCMEC/D3 cells were reseeded in a 96-well plate (Corning, NY, USA) at a density of 1 × 10^4^ cells/well for the CCK-8 assay. To construct the BBB in vitro model for the TEER assay, cells were reseeded at a density of 5.0 × 10^4^ cells/cm^2^ on the membrane of hanging cell culture inserts (0.4 µm pore, 0.3 cm^2^, PET, Millipore, Bedford, MA, USA) in 24-well microplates. For IF and the qRT-PCR assay, cells were reseeded at a density of 2.0 × 10^4^ cells/cm^2^ separately in dishes (35 mm, Labselect, Beijing, China). Cells between passage 8 and 10 were used in this study. The medium was changed every two days until a monolayer was achieved.

### 4.4. CCK-8 Assay

hCMEC/D3 cell suspensions (100 μL, 1 × 10^4^ cells/well) were added to a 96-well plate at 37 °C in a 5% CO_2_ incubator. After corresponding treatments for different groups, cells were treated with 10 µL of CCK-8 solution (AbMole, BioScience, Harvard, TX, USA) at 37 °C for 1 h. The optical density was measured at a wavelength of 450 nm using a microplate reader (Agilent, Santa Clara, CA, USA).

### 4.5. Molecular Docking

AutoDock Tools 1.5.6 was used to simulate the docking of FGFC1 to TJC proteins, including CLDN-5, OCLN, ZO-1, and VE-cad. The three-dimensional structures of these TJC proteins were obtained from the Protein Data Bank (accession number pdbO00501, 1WPA, 4YYX, 2RCE, 3SHU, respectively). FGFC1 was visualized and edited using ChemOffice Professional 19, and subsequently converted into three-dimensional structure files by using the Open Babel GUI software (v2.4.1). The simulated protein docking was performed using the HDOCK SERVER platform, and the results were displayed by using the DiscoveryStudio and PyMOL programs.

### 4.6. TEER Assay

The barrier function was indicated by the tcTEER values, measured by a Millicell ERS-2 system (Millipore, Bedford, MA, USA) in the in vitro model of hCMEC/D3 cells after temperature calibration. The hCMEC/D3 cells successfully formed a monolayer from 6 to 8 days after the reseeding, when the tcTEER values of the model became stable around 21–265 Ω/cm^2^ (310.15 K). Monolayers were pretreated with the FGFC1 solutions (25, 55, 80, 100 μM) from the apical side for 2 h (FS_25, FS_55, FS_80, and FS_100) or not (blank, NC) (37 °C, 5% CO_2_), followed by the administration at the basolateral side of 1 mM H_2_O_2_ (NC) or without (blank) for another 3.5 or 4 h. The tcTEER values of each group were measured every 1 h during the pretreatment duration, and then, every 30 min throughout 3.5 or 4 h incubation with H_2_O_2_ until the tcTEER values became stable.

### 4.7. Immunofluorescent Staining Microscopy 

After the monolayers were pretreated with the FGFC1 solution (FS_25, FS_55, FS_80 and FS_100) or without (blank, NC) for 2 h, 1 mM H_2_O_2_ was administrated (NC) or not (blank) for another 4 h. The monolayers were fixed in paraformaldehyde at 4 °C for 30 min and rinsed three times with Tris-HCl buffer solution with 0.05% Tween-20 (TBST, Servicebio, Wuhan, China) for the immunofluorescent staining microscopy. The monolayers were then blocked with bovine serum album (BSA, 5% (v/v) in TBST) for 30 min at room temperature. Rabbit anti-CLDN-5, OCLN, ZO-1, and VE-cad antibodies were used overnight at 4 °C, followed by the 2nd antibody (FITC isomer goat anti-rabbit IgG antibody) in the dark at room temperature. A fluorescence microscope (BX53F2, Olympus, Tokyo, Japan) was used to observe the expression of each protein. Using ImageJ (version 1.46, NIH), four immunostained photomicrographs were randomly selected from each group and semi-quantitatively measured based on the proportion of target protein-positive areas.

### 4.8. RNA Extraction from hCMEC/D3 Cells and Quantitative RT-PCR

hCMEC/D3 cells underwent incubation with (NC) or without (blank) 1 mM H_2_O_2_ for 2.0, 3.0, and 3.5 h separately right after the 2 h pretreatment with the FGFC1 solutions (55, 80, 100 μM) (FS_55, FS_80, and FS_100); total RNA was extracted from these cells by a SteadyPure Quick RNA Extraction Kit (Accurate, Hunan, China) according to the supplier’s protocol. The quantity and purity of total RNA were measured by using Nanodrop (ThermoFisher, Waltham, MA, USA). Then, cDNAs were synthesized from 1 µg of total RNA using Evo M-MLV RT Mix Kit by gDNA Clean for qPCR Ver.2 (Accurate, Hunan, China). Quantitative RT-PCR was performed in a 20 µL reaction system using the SYBR Green Premix Pro Taq HS qPCR Kit and gene-specific primers (see Supplementary Information). Each mRNA expression was normalized to glyceraldehyde-3-phosphate dehydrogenase (GAPDH) and calculated by the comparative CT method to be expressed as fold-change compared to the blank group.

### 4.9. Statistical Analysis

All the tcTEER values were indicated as relative tcTEER values with reference to their initial values as 100%. Statistical analyses were performed using the GraphPad Prism software (version 7). Statistical significance was determined by the Mann–Whitney U test for the comparison between two groups and the Kruskal–Wallis test was used to analyze the comparisons among multiple groups. All tests were performed 6 times and values are indicated as mean ± SD.

## 5. Conclusions

In this study, deep-sea-derived fibrinolytic compound FGFC1, with a novel thrombolysis mechanism, has been firstly reported to protect TJCs from H_2_O_2_-induced dysfunction via regulating their key factors, including CLDN-5 and VE-cad, mainly at the transcriptional levels; the most efficient concentration is 100 μM. With TJC proteins indicated as the potential targets of FGFC1, this study also provides evidence at the molecular level for FGFC1 reducing the risk of bleeding transformation following its application in thrombolytic therapy for cerebral thrombosis.

## Figures and Tables

**Table 1:** Comparison of the protective effects on the TJC proteins for various concentrations of FGFC1 solution against H_2_O_2_-induced depression.

Mean Integral Optical Density (Relative to the Blank)	NC	FS			
		25 µM	55 µM	80 µM	100 µM
CLDN-5	42.51%	49.10%	67.64%	72.11%	93.29%
OCLN	84.76%	85.29%	83.09%	95.52%	100.37%
ZO-1	89.38%	87.48%	95.75%	96.83%	102.33%
VE-cad	68.91%	67.67%	76.50%	82.42%	105.67%

**Table 2:** The binding positions and energies in optimal conformations.

	FGFC1-CLDN-5	FGFC1-OCLN	FGFC1-ZO-1			FGFC1-VE-cad
			(PDZ1)	(PDZ2)	(PDZ3)	
Hydrogen bonding	TYR158	TYR420, ASN435	SER:57, SER:39, SER:57, ARG:74		LYS87, SER6	ASP86
Electrostatic		HIS499			LYS B:8	LYS48
Hydrophobic	LEU73, PRO158, LYS65, LYS48, TYR68	TRP416, LEU446	LYS109, ARG108, HIS46, VAL55	GLU250, TYR198, LEU202, ILE249	LYS A:8, LYS A:87, GLY45	VAL55, TYR67, LYS48
Binding energy	−6.9 kcal/mol	−6.1 kcal/mol	−7.7 kcal/mol	−5.4 kcal/mol	−5.0 kcal/mol	−7.2 kcal/mol

**Note:** The docking scoring function of AutoDock Vina worked well to rank binding poses. However, the scores are not necessarily related to the binding affinity between ligand and receptor.

## Data Availability

The data presented in this study are available.

## Supplementary Materials

The following supporting information can be downloaded at: https://www.mdpi.com/article/10.3390/md22080341/s1, Table S1: Sequences of primers (human) used for quantitative RT-PCR.
