# Supplementary material for: Protection of Tight Junctional Complexes between hCMEC/D3 Cells by Deep-Sea Fibrinolytic Compound FGFC1
Source: Mar Drugs. 2024 Jul 26;22(8):341. doi: 10.3390/md22080341 (PMC11355241; doi:10.3390/md22080341)
Supplement: Supplementary file 1 [file marinedrugs-22-00341-s001.zip › marinedrugs-3084268-supplementary.pdf]

Table S1. Sequences of primers (human) used for quantitative RT-PCR.

Genes	Forward	Reverse	Product size (bp)	GenBank Accession NO.
	Sequence 5'- 3'	Sequence 5'- 3'		
CLDN-5	GAGGCGTGCTCTACCTGTTT	TAAAACTCGCGGACGACAATG	41	NM_003277
OCLN	AGCGGTTTTATCCAGAGTCTTCC	AGTCATCCACAGGCGAAGTTAAT	46	NM_001205254
ZO-1	CGAGCGATCTCATAAACTTCGT	CCTCGGAAACCCATACCAG	41	NM_003257
VE-cad	ACAAGGACATAACACCACGAA	CCGTGTTATCGTGATTATCCGTGA	45	NM_001795
GAPDH	GCACCGTCAAGGCTGAGAAC	TGGTGAAGACGCCAGTGGA	39	NM_001256799

bp, base pairs; GADPH, glyceraldehyde-3-phosphate dehydrogenase
